# Tobacco and alcohol use, sexual behavior and common mental disorders among military students at the Police Academy, São Paulo, Brazil. A cross-sectional study: Tabagismo e consumo de álcool, comportamento sexual e transtornos mentais comuns em estudantes militares da Academia de Polícia, São Paulo, Brasil. Um estudo transversal

**DOI:** 10.1590/1516-3180.2014.9102711

**Published:** 2014-12-19

**Authors:** Arlene de Maria Perez, Isabela Martins Benseñor

**Affiliations:** I MD. Postgraduate Student, Department of Internal Medicine, Universidade de São Paulo (USP), São Paulo, Brazil.; II MD, PhD. Associate Professor, Department of Internal Medicine, Universidade de São Paulo (USP), São Paulo, Brazil.

**Keywords:** Military personnel, Tobacco, Alcoholic beverages, Sexual behavior, Mental health, Militares, Tabaco, Bebidas alcoólicas, Comportamento sexual, Saúde mental

## Abstract

**CONTEXT AND OBJECTIVE:**

The lifestyle of military personnel has been little studied in Brazil. This study evaluated the frequencies of tobacco and alcohol use, sexual behavior and mental health among military students.

**DESIGN AND SETTING:**

Cross-sectional study at the Police Academy, in São Paulo.

**METHODS:**

Students answered a questionnaire about tobacco use, alcohol consumption, sexual behavior and common mental disorders (CMDs). To analyze associations among the frequencies of smoking and alcohol use, sexually transmitted diseases (STDs) and CMDs during the undergraduate years, we built a multinomial logistic regression model adjusted for age and sex.

**RESULTS:**

All 473 students were invited to participate and 430 (90.9%) agreed (10.5% were women). Most were white (76.6%), aged < 30 years, from the upper middle class (78.1%). The frequency of smoking was 6.5%, alcohol consumption 69.3%, STDs 14% and CMDs 15.6%. The use of condoms was low. Fourth-year students presented a lower odds ratio (OR) for STDs than the first-year students: 0.44 (95% confidence interval: 0.22-0.90). Third-year students presented a lower OR for CMDs than the first-year students.

**CONCLUSION:**

The frequencies of smoking and CMDs were low, while the frequency of alcohol consumption was similar to that of the Brazilian population. The use of condoms was low, in comparison with previous studies with similar samples. The results suggest that there was a certain degree of protection against CMDs and STDs during the undergraduate years.

## RESUMO

**CONTEXTO E OBJETIVOS:**

O estilo de vida dos militares foi pouco estudado no Brasil. Este estudo avaliou frequência de tabagismo, consumo de álcool, comportamento sexual e saúde mental em estudantes militares.

**TIPO DE ESTUDO E LOCAL:**

Estudo transversal na Academia de Polícia, São Paulo.

**MÉTODOS:**

Alunos responderam questionário sobre tabagismo, uso de álcool, comportamento sexual e transtornos mentais comuns (TMC). Para analisar a associação das frequências de tabagismo, uso de álcool, doenças sexualmente transmissíveis (DST) e TMC durante a graduação, realizou-se regressão logística multinomial ajustada por idade e sexo.

**RESULTADOS:**

Todos os 473 estudantes foram convidados e 430 (90,9%) aceitaram participar (10,5% mulheres). A amostra foi composta majoritariamente de brancos (76,6%), com menos de 30 anos, de classe média alta (78,1%). A frequência de fumo foi de 6,5%, de consumo de álcool, 69,3, de DST, 14% e de TMC, 15,6%. O uso de preservativos foi baixo. Estudantes do quarto ano apresentaram uma menor razão de chances (RC) de DST comparados aos do primeiro ano: 0,44 (intervalo de confiança 95%, 0,22-0,90). Estudantes do terceiro ano apresentaram RC menores de ter um TMC comparados aos do primeiro ano.

**CONCLUSÃO:**

As frequências de tabagismo e de TMC foram baixas e a de álcool foi similar à da população brasileira. O uso de preservativos foi baixo comparado a estudos anteriores em amostras semelhantes. Os resultados sugerem uma certa proteção para TMC e DST ao longo da graduação.

## INTRODUCTION

Current military operations create the need for frequent mobilizations and it is imperative that the active forces remain physically and mentally healthy to carry out their military duties.^1^ It is crucial to determine the presence of unhealthy behaviors in the military population in order to prevent potentially catastrophic personal and social events.

Smoking is a major cause of premature deaths that can be prevented worldwide. It relates to heart disease, stroke, chronic obstructive pulmonary disease and several types of cancers. The military are a segment of the population with disproportionately high rates of smoking: above 25%.^2^^,^^3^ Cigarette smoking is very common in the military and it is of particular importance that many soldiers start smoking after entering military life, thus raising the possibility that this is a culture that encourages smoking.^4^ The use of tobacco has declined in Brazil over the last few years, and the average is 11.3% among all the adult population living in state capitals.^5^^,^^6^

Excessive alcohol consumption is common among military personnel on active duty in the United States, and it is strongly associated with adverse health problems with various social consequences.^7^ Because of easy access, and common use within military culture, alcohol is consumed in recreational activities and also to help cope with stressful and traumatic events associated with military duty or combat.^8^ In the United States, 78.5% of active military personnel consume alcohol, and 20% are heavy drinkers.^9^ In Brazil, the prevalence of binge drinking (≥ 5 doses among men and ≥ 4 among women) in the population over 18 years of age was 17.6% (95% confidence interval, CI: 16.8-18.4) in 2011.^10^

Sexually transmitted diseases (STDs) have traditionally been a challenge for military leaders.^11^ Several factors place soldiers at risk of acquiring STDs. The vast majority of soldiers are young sexually active men who are susceptible to peer pressure and often spend long periods of time away from their homes, with restricted opportunities to build long-term relationships. They also have frequent contact with sex workers. Rounding out the problem is the fact that soldiers are used to an overall risk-taking lifestyle, often presenting attitudes of invulnerability that can result in risky sexual behavior.^12^ In 1989, the Canadian armed forces conducted a survey on health and found that 17% of all personnel never used condoms or used them only sometimes.^13^ The percentage of regular condom use has increased substantially among Brazilian military conscripts, from 38% in 1997 to 49% in 2002.^14^

The mental health of any fighting force influences its effectiveness and it has been shown that good mental health is an essential factor for the productivity of military personnel.^15^ Witnessing death, destruction and threats to life may have consequences for their mental health.^16^ Attempts to screen for physical and mental health are not commonly accepted among men, who often lack confidence to discuss psychological problems with the staff on duty.^17^

There are few studies on the military population in Brazil and this is the first study concerning the lifestyle and behavior of the future commanders of the Military Police of São Paulo. The Military Police of São Paulo is a military organization responsible for ostensive policing and preservation of public order, with no relationship with the Brazilian Army. It has in its ranks 81,000 soldiers led by around 5,000 officers (Board staff, Personnel Map, May 30, 2014) who are trained at the Academy of Military Police, in Barro Branco. These student officers will become commanders and role models for their subordinates in one of the most stressful professional activities that exists.

## OBJECTIVE

Our objective was to analyze the frequencies of tobacco and alcohol use, sexual behavior and common mental disorders among military students according to gender, degree year and duration of military life.

## METHODS

This was an observational, cross-sectional study, on students attending the officer training course at the Academy of Military Police, in Barro Branco, state of São Paulo. The course lasts four years. These students underwent a selection process through the Foundation for University Entrance Examinations (Fundação Universitária para o Vestibular, Fuvest), a body that is responsible for selecting students who qualify to attend a public university in the state of São Paulo (selection is made in accordance with the results from tests that encompass questions on several areas of knowledge). After this selection, students are tested for physical and psychological fitness prior to joining the military. Most are male, 25 years of age or less, and from the state of São Paulo.

This study was authorized by the Department of Education of the Military Police of São Paulo, in collaboration with the commander of the Military Police Academy. The volunteer participants signed a consent form, which was approved by the Institutional Ethics Committee of the University Hospital in accordance with Resolution CNS 196/96. All students at the Military Academy were invited to participate in this study after being informed of its purpose and about the questionnaire that would be applied. It was explained to the students that their participation would be voluntary, and that they had the right to withdraw from the study at any time. The questionnaires were explained to the students in the presence of someone who had been trained to answer possible queries. They were given a time period of one weekend to complete the questionnaire. Thus, the questionnaires were handed out to the students on September 10, 2010, and were returned on September 13, 2010. Once answered, the questionnaires were sealed by the students themselves in an envelope without any identification. All the envelopes were left in a specific location and then collected by the researcher.

The questions asked for information on sociodemographic variables, general health, alcohol use, tobacco use, sexual behavior and common mental disorders. The sociodemographic questionnaire asked for information about age (years), sex, race, origin (the state capital or elsewhere), marital status (single, married, widowed or divorced) and history of previous military service (armed forces or military police).

Alcohol consumption was assessed by means of a questionnaire that was used in the Brazilian Longitudinal Study of Adult Health (ELSA-Brasil). This asked for information about previous use of drinks, type of beverage (white wine, red wine, beer or spirits) and the amount ingested weekly. Because this was a work environment and a military service, we did not apply any questions about binge drinking or dependence.^18^^,^^19^

Smoking was defined as a minimum consumption of 100 cigarettes (five packs) over the course of the individual’s life and was assessed in terms of the age at which smoking started, current consumption (daily amount), years of smoking (removing time spent without smoking), living with people who smoked (at home or at work) and change in consumption pattern after entering military life.^18^

We used a specific questionnaire to assess sexual behavior, including questions about the age at which sexual life started, partnerships (regular, regular and casual or only casual), sexual contact with sex workers (frequent, occasional or never), use of condoms (always, mostly, seldom or never), and previous treatment for sexually transmitted diseases. We used the questionnaire that was applied by the Ministry of Health to Brazilian army conscripts in 1997-2002, with authorization from the Ministry of Health^14^

To assess mental health, we used the Self-Report Questionnaire with 20 questions (SRQ-20), which makes it possible to identify common psychiatric mental disorders at primary care. This was developed by Harding et al. for the World Health Organization (WHO),^20^ and was validated by Mari and Williams for use in Brazil.^21^ It identifies common mental disorders (CMDs), which are conditions that, although not involving a formal psychiatric diagnosis, indicate significant psychiatric distress. For a participant to be considered as a possible case, the cutoff point was five/six for men (sensitivity 89% and specificity 81%) and seven/eight for women (sensitivity 86% and specificity 77%). For the present analysis, the cutoffs were taken to be 6 and 7 for men and women, respectively.^21^

### Statistical analysis

Categorical variables were expressed as percentages and compared using the chi-square or Fisher exact test, as appropriate. Continuous variables were expressed as means and their standard deviations and were compared using ANOVA (analysis of variance) with the Bonferroni *post hoc* test. A test for trend was used as appropriate.

A multinomial logistic regression model was built to evaluate associations between increasing frequency of risk factors for sexually transmitted diseases and common mental disorders and the academic year of the degree course (first year to fourth year), using the first year as the reference. Odds ratios (OR) were presented in a crude form and were then adjusted for age and gender, for the whole sample. Since mean age increased from the first year to the fourth year, we adjusted for age, since lifestyles could change and presence of disorders could increase as consequences of ageing but not of time at the military academy. Furthermore, we restricted the analysis to men (since the number of women was small) and we also stratified the male sample into those who had worked as military policemen before entering the Police Academy and those who had never worked as military policemen before. All these analysis were presented in a raw format and were adjusted only for age.

The significance level was set at 5%. All analyses were performed using SPSS 16.0.

## RESULTS

After the research objectives had been presented to the original group of 473 student officers, 430 (90.9%) agreed to participate and gave their informed consent. In relation to sociodemographic data, our sample included 384 men and 45 women (one individual did not declare the sex). Most of them were white (76.2%), under 30 years of age, unmarried, upper middle class (78.1%), from the state of São Paulo (99.1%) and were supporting themselves (75.5%) (Table 1).

**Table 1.**
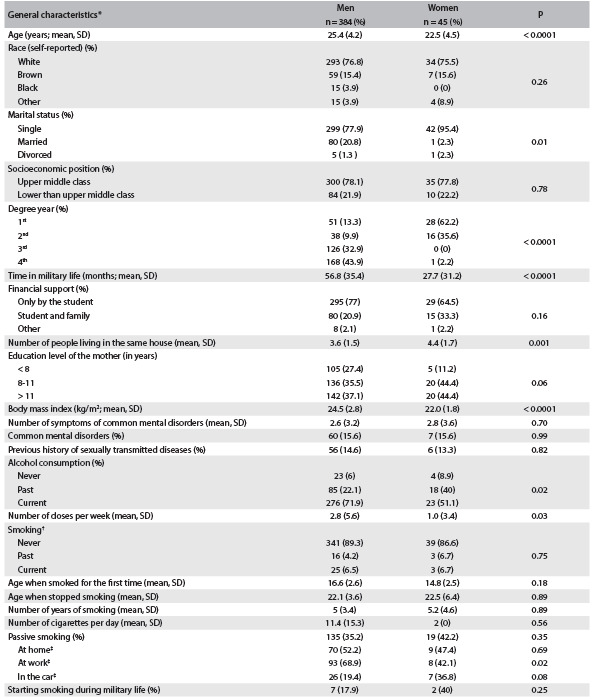
General characteristics of the sample according to gender

SD = standard deviation. *Since one student did not answer the question about sex, the data in this table are restricted to 429 participants with information about sex; ^†^Percentage calculated only for students who reported current or past smoking (N men/women = 41/6); ^‡^percentages calculated only for students who reported passive smoking (N men/women = 135/19).

Considering personal habits, it was found that 88.3% of the students had never smoked and only 6.5% were current smokers, with no statistically significant difference between the genders. Regarding alcohol consumption, 69.7% of the students reported drinking alcohol (71.9% of men and 51.1% women), with a statistically significant difference between the genders (Table 1). We found that mental disorders were present in 15.6% of the sample (no gender difference) (Table 1). Most of the students who were past or current smokers had started to smoke before military life.

Regarding sexual behavior, 97.7% of the men and 77.8% of the women reported that sexual initiation had occurred, with age at onset of 16.4 (standard deviation, SD: 2.0) years for men and 17.6 (SD: 2.6) years for women, with a statistically significant difference between the men and women (P = 0.001). Protection with a condom at the first intercourse was used by 76.3% of the men and 88.6% of the women (P = 0.10), but at the last sexual intercourse only 46.1% of the men and 62.9% of the women protected themselves (P = 0.06). The average number of regular partnerships over the last 12 months was 1.3 (SD ± 1.7) for the men and 1.1 (SD ± 0.6) for the women (without any statistically significant difference); 17.4% of the men and 36% of the women protected themselves during all intercourse with regular partners over the last 12 months. Among the students who had had casual partners over the last 12 months, we observed that there were an average of 2.2 (SD: 5.4) partnerships among the men and 0.4 (SD: 1.3) among the women and that only 64.2% of the men and 60% of the women protected themselves at all times in these partnerships. Only 14 men (3.7%) had paid for sex and 92.9% of them protected themselves at all times (Table 2). We found that 14.6% of the men and 13.3% of the women reported a previous history of sexually transmitted diseases (no statistical difference between the genders) (Table 2).

**Table 2.**
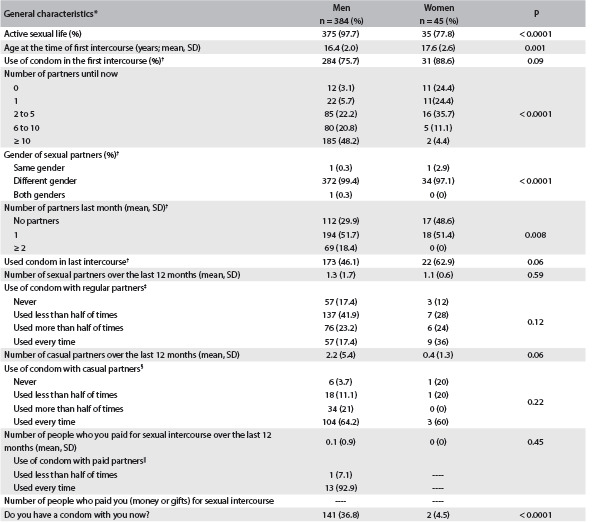
Characteristics of sexual behavior according to gender

SD = Standard deviation. *Since one student did not answer the question about sex, the data in this table are restricted to 429 participants with information about sex; ^†^percentages calculated only for participants reporting active sexual life (N men/women = 375/35); ^‡^percentages calculated only for participants reporting regular partners (N men/women = 327/25); ^§^percentage calculated only for participants reporting casual partners (N men/women = 162/5); ^||^percentages calculated only for participants reporting paid partners (N men/women = 14/0).

Table 3 shows the lifestyle changes according to the degree year. It was observed over the degree years that there was an increase in the mean number of symptoms of common mental disorders from the first to the fourth year (2.2 in the first year vs. 3.0 in the fourth year; P = 0.06; P for trend 0.02). Although the increase was statistically significant, it had low clinical relevance and the frequency of common mental disorders did not increase over the period. There was no statistically significant increase in the frequency of current smokers over the years at the Academy (P = 0.37), or in the frequency of current alcohol consumption (P = 0.87) (Table 3).

**Table 3.**
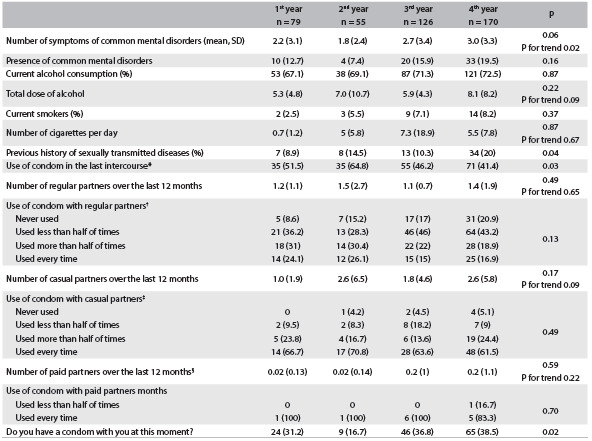
Changes in lifestyle according to year in the degree course

SD = Standard deviation. *percentages calculated only for participants reporting active sexual life (N 1^st^, 2^nd^, 3^rd^ and 4^th^ years = 68, 54, 119 and 169, respectively); ^†^percentages calculated only for participants reporting regular partners (N 1^st^, 2^nd^, 3^rd^ and 4^th^ years = 58, 46, 100 and 148, respectively); ^‡^percentages calculated only for participants reporting casual partners (N 1^st^, 2^nd^, 3^rd^ and 4^th^ years = 21, 24, 44 and 78, respectively); ^§^percentages calculated only for participants reporting paid partners (N 1^st^, 2^nd^, 3^rd^ and 4^th^ years = 1, 1, 6 and 6, respectively).

The numbers of regular, casual and paid sexual partners over the last 12 months did not change over the academic years (respectively P = 0.49, P = 0.17 and P = 0.59). The use of protection in these relationships did not change over the years (respectively P = 0.13, P = 0.49 and P = 0.70). Among those who protected themselves in the last sexual intercourse, we found that first and second-year students protected themselves more than third and fourth-year students (51.5% in the first year and 64.8% in the second year versus 46.2% in the third year and 41.8% in the fourth year; P = 0.03). However, when students were asked whether they had had condoms at the time of sexual intercourse, the third and fourth-year students responded affirmatively more often than the first and second-year students (31.2% in the first year, 16.7% in the second year, 36.8% in the third year and 38.5% in the fourth year; P = 0.02) (Table 3).

Table 4 describes the results from the multinomial logistic regression models for all samples. We restricted our analysis only to men, and a further analysis was performed including only the men who belonged to the military before admission to the Police Academy and only the men who had never belonged to the military before admission to the Police Academy (data not shown). For all samples, logistic models found lower odds ratio for associations with presence of common mental disorders among third-year students, compared with those in the first year (reference group), even after adjustment for age and sex: OR, 0.27; 95% CI, 0.08-0.86 (Table 4). For all the men and also only among those who had belonged to the military before admission to the Police Academy, we also found a protective OR for the third-year students, compared with the first-year students (reference group) after adjustment for age (data not shown). For all samples, a lower OR for the association between the presence of sexually transmitted diseases was observed in the fourth year, compared with the first year: OR, 0.45; 95% CI, 0.22-0.91 (Table 4). For all the men and also only for the men who had belonged to the military before admission to the Police Academy (data not shown), a protective effect was detected among the fourth-year students, in comparison with the reference group. No protection against sexually transmitted diseases or common mental disorders was found among men who had never belonged to the military before admission to the Police Academy.

**Table 4.**
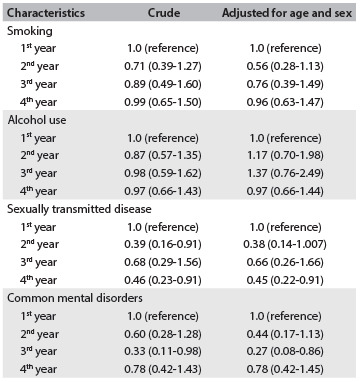
Odds ratios (with 95% confidence intervals) for the associations between smoking, alcohol consumption, sexually transmitted diseases, common mental disorders and the degree year at the Police Academy

## DISCUSSION

In this cross-sectional analysis, the frequency of smoking among the students was 6.5% for men and 6.7% for women. Most of the students who smoked had started before beginning at the Police Academy. Current alcohol consumption was higher among the men than among the women (71.9% among men versus 51.1% among women; P = 0.03), as also was the number of doses per week (2.8 among the men versus 1.0 among the women; P = 0.03). Regarding use of condoms during all intercourse with regular, casual and paid partners, the frequencies for men were 17.4%, 64.2%, and 92.9%, respectively; and for women 33.3%, 60% and none of the condom users reported paying for partners. Around 14% in both genders had a self-reported history of previous sexually transmitted diseases. The frequency of common mental disorders was 15% for both genders. The number of symptoms of these problems increased over the degree course, but the frequency of common mental disorders did not increase over time. The most important results from the multinomial logistic regression models were that there were protective effects against common mental disorders among third-year students, compared with the reference group; and against sexually transmitted diseases among fourth-year students, compared with first-year students, for all of the sample, for all men and for only the men who had previously belonged to the army. These findings suggested that there may have been an increase in the protection with increasing degree year.

In our sample, we found that 6.5% of the student officers were current smokers. This differs from previous Brazilian data obtained through the Brazilian Survey of Risk Factors for Chronic Diseases, which was conducted over the telephone and found a proportion of 11.3% (14.4% among men and 8.6% among women);^5^^,^^6^ and from data on the United States population, in which the proportion was 19.8% in 2007.^22^ Among the American military, smoking prevalence greater than 30% has been reported among active-duty military personnel.^23^ Considering younger officers with characteristics similar to those of our sample, the prevalence of smokers was reported to be 11.2% in a study conducted from 2005 to 2008.^23^ Our results depicted lower frequencies of smoking compared with the tobacco usage reported among a national sample of college students in Brazil, which were was follows: lifetime prevalence of 51.7% among men and 42.9% among women; prevalence over the last 12 months of 31.8% among men and 24.8% among women; and prevalence over the last month of 23.5% and 20.1% among men and women, respectively.^24^ Over the year-groups at the Academy, we found an increase in the percentage of smokers. Although not significant, this was similar to previous published data, showing an increase in smoking among older people,^2^ thus suggesting that this military culture facilitated smoking. However, in our data, most of the smokers began smoking before entering military life and, moreover, the results from the multinomial logistic regression did not detect any change in tobacco usage according to the number of years until graduation.

In relation to alcohol consumption, we found that 69.7% of the students (71.9% of the men and 51.1% of the women) consumed some sort of alcoholic drink. These frequencies are similar to data from the Brazilian population survey conducted by the Brazilian Information Center on Psychotropic Drugs (Centro Brasileiro de Informações sobre Drogas Psicotrópicas, CEBRID), which reported that the alcohol consumption rate was 68.7% over the lifetime, with no changes in different age groups.^25^ Our data are also similar to American data showing that the consumption rates were 75.2% among young pre-conscription individuals and 76% among active-duty military personnel; 19.8% of the latter group reported heavy consumption.^9^ In our sample, we did not include any question to assess alcohol abuse or binge drinking, in order to prevent negative answers, since this was a survey conducted in the workplace in a military working population. However, our frequency was lower than the results from a Brazilian study that evaluated the prevalence of alcohol use among a sample of college students. The lifetime prevalence of alcohol consumption was 90.3% among the men and 83.1% among the women; the prevalence over the last 12 months was 77.3% among the men and 68% among the women; and the prevalence over the last month was 66.6% and 55.8% among the men and women, respectively.^24^ Over the year-groups at the Academy, alcohol consumption remained unchanged, thus suggesting that there was no culture that discouraged alcohol intake.

Regarding sexual behavior, our results showed that 17.4% of the men and 33.3% of the women used condoms consistently (in all sexual intercourse) over the last 12 months with regular partnerships, and 43.1% of the men and 62.9% of the women reported that they had protected themselves during the last intercourse. Indeed, we found that 14.5% of the students reported having previously had a sexually transmitted disease. In a study conducted among Brazilian army conscripts in 1998 (taking into consideration the population segment in Rio de Janeiro and São Paulo with completed high school education, which was similar to our sample), it was found that 51.5% of the subjects were condom users in all sexual intercourse with regular partners over the last year, and that 72.6% reported using a condom during the last intercourse. Regarding casual partnerships, the numbers showed that 62.3% of the men and 60% of the women used condoms consistently, while among the conscripts in the Brazilian army, 69.1% protected themselves in all intercourse with casual partners over the last year. Regarding paid partnerships, it was reported that 92.9% of the students who had paid for sex protected themselves on every occasion over the last year, compared with 91.4% among Brazilian conscripts. The percentage of students who protected themselves in regular partnerships was lower in our sample than in the Brazilian military population in regular partnerships. However, for casual and paid partnerships, our results were similar to previous data from Brazil. The prevalence of sexually transmitted diseases reported in the present study was higher than the prevalence of 7.7% found among military conscripts.^26^ The lower frequency of condom use in our sample was associated with higher frequency of STDs, even considering that the lower frequency of usage was concentrated in regular partnerships.

Another study on Brazilian army conscripts in 2007 showed that 38.2% of the sample used condoms consistently with regular partnerships, 53.5% with casual partnerships and 68.1% with paid partners over the last twelve months. Moreover, 13.1% reported having previously had sexually transmitted diseases, thus achieving similar numbers to ours. However, that study did not differentiate the segment between Rio de Janeiro and São Paulo; nor did it separate the young people (so that young men without high school education were analyzed together with those that had had high school education). Thus, it was a sample of young Brazilians that differed from our sample.^27^

In a study on a sample of 351 members of the Defense Force of Belize, mostly composed of men with high school education who were in a stable relationship (mean age = 29 years), it was found that 12% of the sample reported having previously had sexually transmitted diseases.^28^ Despite the differences between the samples, such that the individuals in the Defense Force had a greater mean age and had been in military service for more years than our sample, the result was similar.

In another study on 498 military personnel (composed mainly of unmarried men, of mean age 31 years, who had not completed their high school education) on the border of the Dominican Republic, over 60% of the respondents (51% of the sample) who had had non-monogamous sex over the past 12 months were found to have only made inconsistent use of condoms.^29^ Among a sample of unmarried men in the United States Marines stationed on ships in the western Pacific, of mean age 22 years, with high school education, inconsistent use of condoms was reported in 80%.^30^ Compared with these data, our students showed higher frequency of condom use.

The results from the multinomial logistic regression suggested that there was protection against STDs and CMDs after adjustment for age and sex, for all of the sample; and after adjustment only for age considering all the men or only the men who had belonged to the army before admission to the Police Academy. However, it is impossible to know whether this protection might be verifiable in future years.

Considering the cutoffs in the SRQ-20, i.e. 6 for men and 7 for women, we found that CMDs were present in 15.6% of the students. In a study on the military population in the United Kingdom in which the Patient Health Questionnaire (PHQ) was applied, the prevalence of CMDs among the solders was found to be 27.2%.^15^ In this case, in addition to the difference in the questionnaire used to evaluate mental health, the population studied included military personnel at the time of the war in Iraq, which was different from our sample of soldiers who were not involved in any current military action, thereby making it difficult to correlate the results. The prevalence of CMDs among the American military was 18.3% using the Primary Care Evaluation of Mental Disorders in the form of the Patient Health Questionnaire (PHQ).^30^ Although that study used a different questionnaire, both questionnaires evaluated mental disorders within primary care and the results from the American military were very similar to the prevalence of CMDs in our sample.

Another study conducted in Rio de Janeiro, on 1458 civil policemen and 1108 military policemen, showed that there was high psychological distress (psychosomatic symptoms, depression and anxiety) among military policemen (33.6%) in comparison with civil policemen (20.3%). Although both studies used the SRQ-20, we evaluated a sample of students and the other study evaluated an operational sample of military policemen. Therefore, the higher frequency of CMDs in Rio de Janeiro, compared with ours, would be expected.^31^ Maragno et al. evaluated the prevalence of common mental disorders using SRQ-20 in a population sample in the northern zone of the city of São Paulo, among among subjects aged 15 years or over. They found a prevalence of CMDs approximately 25% higher than the frequency in our sample. However, the differences in age strata and socioeconomic level between the two samples were very large and could explain the different results.^32^

We found that the number of symptoms of CMDs was higher only among students who had still not participated in military operations. However, there was no increase in the frequency of CMDs among them from the first to the fourth year.

Our study has some limitations. Our sample included students at the Military Police Academy who had not yet been involved in military operations. Therefore, comparisons with other military forces in full operation are limited, as well as comparisons with samples from the general population. Few studies included women; therefore, most of the comparisons were made only with the subsample of men. We compared the frequencies of several lifestyle characteristics from the first to the fourth year. However, we did not follow the same cohort of students from the first to the fourth year. Our study evaluated a special population under military orders, asking about sensitive issues. Therefore, all the information was collected anonymously, thus preventing follow-up on specific students until their graduation.

## CONCLUSIONS

We found lower prevalence of smoking in comparison with the prevalence reported among the United States military population, and it was also lower than the prevalence of smoking among Brazilian and American civilians. In relation to alcohol consumption, our data are similar to the frequency of consumption among the Brazilian population and close to that of the United States military population. Using condoms consistently with regular partnerships was less frequent than with casual partners and with paid intercourse. However, even for paid partners, the frequency of condom use was lower than expected, and this probably explains the higher frequency of STDs reported by the students. We found that CMDs were present in 15.6% of the sample, with an increase in the number of symptoms from the first to the fourth year. Logistic regression models did not show any change in the odds relating to smoking and alcohol over the duration of the course, but this could suggest that there is a protective effect against CMDs and STDs during the undergraduate years.
